# Utilizing multiple diffusion metrics in evaluation of corticospinal tract injury in patients with glioblastoma

**DOI:** 10.3389/fnins.2025.1605786

**Published:** 2025-07-28

**Authors:** Ting Chen, Peipei Wang, Eryuan Gao, Jie Bai, Guohua Zhao, Kai Zhao, Gaoyang Zhao, Yu Zhang, Yong Zhang, Mengzhu Wang, Guang Yang, Boyan Xu, Xiaoyue Ma, Jingliang Cheng

**Affiliations:** ^1^Department of Magnetic Resonance Imaging, The First Affiliated Hospital of Zhengzhou University, Zhengzhou University, Zhengzhou, China; ^2^MR Research Collaboration, Siemens Healthineers Ltd., Beijing, China; ^3^Shanghai Key Laboratory of Magnetic Resonance, East China Normal University, Shanghai, China; ^4^MR Research, GE Healthcare, Beijing, China; ^5^Tianiian Laboratory of Advanced Biomedical Sciences, Zhengzhou, China

**Keywords:** diffusion MRI, diffusion kurtosis imaging, glioblastoma, corticospinal tract injury, motor function

## Abstract

**Objectives:**

This study aimed to evaluate the efficacy of diffusion tensor imaging (DTI), diffusion kurtosis imaging (DKI), neurite orientation dispersion and density imaging (NODDI), and mean apparent propagator-magnetic resonance imaging (MAP-MRI) in detecting CST injury caused by GBM and to compare their performances.

**Materials and methods:**

We enrolled 76 patients diagnosed with GBM with motor weakness (MW, *n* = 22) or normal motor (NM, *n* = 54). Bilateral CSTs were reconstructed, and a comparative analysis of diffusion parameters was performed based on four imaging models between affected and healthy sides. Relative diffusion parameters were assessed in the MW and NM groups. Statistical analyses were performed using SPSS software.

**Results:**

Significant alterations in most diffusion parameters of DTI, DKI, NODDI, and MAP-MRI were observed in the affected CST group compared to the healthy CST group (*p* < 0.05). Notable differences in the relative diffusion parameters were observed between the MW and NW groups across all four imaging models (*p* < 0.05). Specifically, DKI-based relative mean kurtosis (MK) exhibited a higher area under the curve (0.813), demonstrating greater sensitivity and specificity, which significantly positively correlated with muscle strength. DeLong^’^s test revealed a significant performance difference between DKI and DTI.

**Conclusion:**

Diffusion parameters from DTI, DKI, NODDI, and MAP-MRI are useful for evaluating CST injury. While DKI-derived MK and NODDI-derived ICVF achieved identical high AUC values, MK exhibited a more balanced sensitivity-specificity profile for assessing microstructural alterations in CST injury, this advantage of DKI may better address clinical demands, potentially aiding in surgical planning and preserving motor function in patients with GBM.

## Introduction

Gliomas, originating from glial cells, are the most common primary brain tumors (Brown et al., 2016; Przybylowski et al., 2021). Notably, glioblastoma (GBM), a severe subtype of gliomas, tends to invade white matter fibers (Cruz et al., 2022). Among these, the corticospinal tract (CST) stands out as a vital neural pathway for motor function, governing the voluntary motion of limbs. CST injury can lead to motor dysfunction (Lemon, 2008). Despite surgical resection remains the cornerstone of glioma management and the extent of resection is a major prognostic factor, it is critical to recognize that preserving patients’ quality of life and long-term survival depends on avoiding postoperative motor deficits (Bush et al., 2017; Sales et al., 2022). Resection of gliomas in or close to motor areas carries a high risk of surgery-related deficits. Therefore, preoperative assessment of CST involvement is essential for surgical planning to mitigate surgical damage to this crucial pathway and to strike a balance between maximum tumor removal and preserving function (Gunnarsson et al., 2002; Kreth et al., 2013).

Over the past decade, diffusion magnetic resonance imaging (dMRI) has become a crucial tool for assisting surgical planning. Diffusion tensor imaging (DTI) has gained widespread clinical application in non-invasively detecting microstructural alterations in the CST (Jeurissen et al., 2019; Yang et al., 2021; Henderson et al., 2020). Notably, fractional anisotropy (FA) is a biomarker for the integrity of white matter fibers, with a reduction in FA correlating with CST injury resulting from brain tumors (Bucci et al., 2013; Essayed et al., 2017). However, DTI relies on the assumption that water molecules conform to a Gaussian distribution (Jelescu et al., 2016). Given that DTI only assesses a single diffusion direction within each voxel, it frequently fails to resolve cross/kissing fibers and other intricate fiber structures (Fernandez-Miranda et al., 2012; Bopp et al., 2021). The limitation leads to an underestimation of FA in regions containing crossing fibers compared to those without, potentially resulting in an inaccurate assessment of compromised fiber structural integrity—a significant challenge for clinicians (Oouchi et al., 2007).

The development of non-Gaussian models for dMRI has substantially overcome the limitations inherent in DTI, thereby enabling a more precise characterization of the tissue microstructure. Diffusion kurtosis imaging (DKI), serving as an extension of DTI, provides a more comprehensive understanding of tissue microstructure (Steven et al., 2014; Jensen et al., 2005). DKI outperformed DTI in delineating subtle alterations in white matter integrity, as evidenced in the study of patients with amyotrophic lateral sclerosis (Anand et al., 2023). Furthermore, neurite orientation dispersion and density imaging (NODDI), a novel tissue-specific compartment model, has been developed to uncover mechanisms of white matter integrity impairment unexplained by FA and to quantify microstructural changes with heightened specificity (Zhang et al., 2012). NODDI serves as a complementary tool to DTI, providing additional biological insights into the microstructural integrity of the CST in children with unilateral cerebral palsy (Nemanich et al., 2019). When applied to investigate CST pathology in patients with high-grade gliomas, NODDI has exhibited performance comparable to DTI (Jiang et al., 2021). Mean apparent propagator-magnetic resonance imaging (MAP-MRI), another recently introduced diffusion model based on the q-space information, can calculate the probability density function of molecular displacement and comprehensive quantitative indicators, potentially offering a more sensitive imaging biomarker (Ozarslan et al., 2013; Avram et al., 2016). Jiang et al. claimed that MAP-MRI was an effective approach for evaluating microstructural changes in CST injury (Jiang et al., 2021). Collectively, the aforementioned studies underscore the immense promise of non-Gaussian models in characterizing the microstructure of tissues.

However, to date, research on exploring non-Gaussian models for assessing CST injury in patients with glioma, especially within the context of a small sample, has been limited. Primarily, these studies have focused on individual non-Gaussian models, with a paucity of research dedicated to CST injury in GBM. Therefore, this study aimed to evaluate the application of different diffusion models (DTI, DKI, NODDI, and MAP) in CST injury induced by GBM and compare their performance to provide valuable clinical guidance.

## Materials and methods

### Study participants

This retrospective study was approved by scientific research and clinical trial ethics committee of the first affiliated hospital of Zhengzhou university (2019-KY-231), and informed consent was waived. Between April 2019 and June 2023, patients with a pathological diagnosis of GBM adjacent to or located in the CST pathway were included in this study. Destruction to the CST may decrease muscle strength. Neurosurgeons utilized the British Medical Research Council grading (MRC) system for motor function assessment, which quantitatively evaluated muscle strength on a scale ranging from 0 to 5 (Dyck et al., 2005). The value of 0 represents no muscle contraction and 5 indicates normal muscle strength. The MRC grading system, as a validated and reliable instrument for motor strength assessment, has been extensively adopted in clinical neurosurgical practice. The weakest limb strength assessment in each patient was used for statistical analysis in this study. Other clinical characteristics, including age, sex, Karnofsky performance status (KPS), tumor location and tumor size were recorded. Inclusion criteria consisted of (Brown et al., 2016) pathologically confirmed GBM according to the World Health Organization 2021 classification criteria, (Przybylowski et al., 2021) age ranging from 18 to 75 years, and (Cruz et al., 2022) acquisition of diffusion-weighted MRI obtained within 2 weeks before surgery and antitumor treatment. Exclusion criteria included: (Brown et al., 2016) prior biopsy or antitumor therapy before MRI examination, (Przybylowski et al., 2021) presence of obvious artifacts or motion artifacts in MR images, and (Cruz et al., 2022) larger tumors involving bilateral CSTs. Finally, 76 patients with GBM, including those with motor weakness (MW; *n* = 22) and normal motor function (NM; *n* = 54), were enrolled in this study.

### MR imaging acquisition

All images were acquired using a 3 T MR scanner (MAGNETOM Prisma, Siemens Healthineers, Erlangen, Germany) equipped with a 64-channel head and neck coil. Structural MRI protocols included T1-weighted imaging (T1WI), T2-weighted imaging (T2WI), axial T2 dark-fluid imaging, and contrast-enhanced axial/sagittal/coronal T1W imaging. The acquisition parameters for diffusion-weighted imaging (DWI) using single-shot echo-planar imaging were as follows: TR = 3,700 ms, TE = 72 ms, matrix = 110 × 110, number of slices = 100, slice thickness = 2.0 mm, FOV = 220 mm × 220 mm, 99 diffusion directions, and 10 different *b*-values (*b* = 0, 350, 650, 1,000, 1,350, 1,650, 2000, 2,650, 2,700, 3,000 s/mm^2^). The acquisition time for DWI was 6 min 37 s.

### Image processing and analysis

Eddy current and motion corrections were applied to all diffusion-weighted images using the Diffusion Kit Eddy tool^1^ (Xie et al., 2016). Subsequently, parameter fitting for DTI, DKI, MAP-MRI, and NODDI was conducted using NeuDiLab software developed in Python, based on the open-sourceDIPY (diffusion imaging in the Python) tool^2^ (Garyfallidis et al., 2014). DTI parameters included axial diffusivity (AD), FA, mean diffusivity (MD), and radial diffusivity (RD); DKI parameters included axial kurtosis (AK), radial kurtosis (RK), mean kurtosis (MK); MAP parameters included mean squared displacement (MSD), non-Gaussianity (NG), NG axial (NGAx), NG radial (NGRad), Q-space inverse variance (QIV), return to the origin probability (RTOP), return to the axis probability (RTAP), return to the plane probability (RTPP); NODDI parameters included intracellular volume fraction (ICVF), isotropic volume fraction (ISOVF) and orientation dispersion index (ODI).

Bilateral corticospinal tracts (CSTs) were reconstructed in DSI Studio^3^ using generalized *q*-sampling imaging (GQI) with a diffusion sampling length ratio of 1.25. The GQI algorithm was selected for its superior capability in resolving complex fiber geometries (e.g., crossing fibers) near pathological regions compared to diffusion tensor imaging (DTI). Preprocessed diffusion data (eddy-current and motion-corrected) underwent fully automated tractography with default parameter. To ensure anatomical fidelity, all automated CST reconstructions underwent rigorous quality assessment by two independent neuroradiologists (20 and 10 years of experience). They evaluated tract continuity, spatial alignment with neuroanatomical landmarks, and absence of aberrant streamlines violating CST topography. Discrepancies (observed in <5% of cases) were resolved by consensus; no manual corrections were applied to retain algorithmic objectivity. The positional relationship between the affected CST and tumor was also recorded. The CST was considered to be located within the CST pathway if the distance to the tumor/infiltrating edema was zero; otherwise, it was classified as being located near the CST pathway. The displacement of the ipsilateral CST (affected side) in relation to the tumor was measured based on the position of the contralateral CST (healthy side) at the level of the maximum tumor size (Supplementary Figure S1). Subsequently, the values of the AD, FA, MD, RD, AK, RK, MK, MSD, NG, NGAx, NGRad, QIV, RTOP, RTAP, RTPP, ICVF, ISOVF, and ODI along the bilateral CSTs were calculated. To relieve the impacts of age and inter-individual differences on diffusion parameters, the relative values of these parameters were computed as the ratios of the metric values of the affected CST to those of the healthy CST. In this study, CST displacement in the MW group ranged from 5.17 mm to 21.80 mm. In contrast, in the NM group, it ranged from 1.50 mm to 21.00 mm; given that the CST displacement of 17 patients in the NM group was less than the minimum value observed in the MW group (5.17 mm), a subsequent subgroup analysis was conducted to exclude confounding bias. This analysis focused on patients (*n* = 37) with CST displacements ≥5.17 mm, following Jiang et al.’s methodology (Jiang et al., 2021).

### Statistical analysis

All statistical analyses were performed using SPSS (version 29.0; SPSS Inc., Chicago, IL, USA) and MedCalc software (version 22.001; MedCalc Software Ltd., Ostend, Belgium). The normality of continuous data was assessed using the Shapiro–Wilk test. Data were presented as mean ± standard deviation (SD) or median (interquartile range). The Mann–Whitney U test was used to compare age, Karnofsky performance status (KPS), tumor size, and CST displacement between the groups. The chi-square test was used to compare sex, tumor location, and the positional relationship between the ipsilateral CST and the tumor. Paired *t*-tests or Wilcoxon tests were used to compare the diffusion parameter values between the affected and healthy CST. The relative diffusion parameter values of the CST were compared between the MW and NM groups using the Mann–Whitney U test or independent *t*-test. All *p*-values were corrected for multiple comparisons via the Benjamini-Hochberg procedure to control the false discovery rate (FDR). Receiver operating characteristic (ROC) analysis was used to evaluate the performance of the relative diffusion parameter values of the CST between the MW and NM, and DeLong^’^s test was used to compare their performance. The Spearman’s correlation was used to examine the association between the relative diffusion parameter and muscle strength assessed by MRC system. A significance level of *p* < 0.05 was set for statistical significance.

## Results

### Patient demographics

A total of 76 patients were enrolled in this study, with 22 clinically evaluated as having motor weakness, while the remaining 54 exhibited normal motor function. The clinical data are presented in Supplementary Table S1. There were no significant differences in age, sex, tumor location, or positional relationship of ipsilateral CST and tumors between the MW and NM groups (*p* > 0.05). However, the KPS score was significantly lower in the MW group than in the NM group, and a more pronounced CST displacement was observed in the MW group (*p* < 0.05).

### Comparison of CST diffusion parameters between affected and healthy sides

In the MW group, all CST diffusion parameters, except ISOVF (*p* = 0.168) and ODI (*p* = 0.528), significantly differed between the affected and healthy sides. Compared with the healthy side, the affected side exhibited a significant increase in AD, MD, RD, MSD, QIV (all *p* < 0.05), and a noteworthy decrease in FA, AK, MK, RK, NG, NGAx, NGRad, RTAP, RTOP, RTPP, ICVF (all *p* < 0.05). In the NW group, significant differences were found only for AD, AK, MK, RK, and ICVF between the affected and healthy sides (all *p* < 0.05). Detailed results are presented in Table 1.

**Table 1:** Comparison of CST diffusion parameters between affected and healthy sides.

Diffusion parameters	MW (*n* = 22)				NM (*n* = 54)			
	Affected CST (*n* = 22)	Health CST (*n* = 22)	t/Z	*p*	Affected CST (*n* = 54)	Health CST (*n* = 54)	t/Z	*p*
AD (10^−3^ mm^2^/s)	1.580 (1.038–1.951)	0.925 (0.873–1.917)	−2.224	0.026*	0.905 (0.872–0.971)	0.887 (0.855–0.929)	−2.286	0.022*
FA	0.310 ± 0.067	0.376 ± 0.073	−3.024	0.006*	0.377 (0.342–0.408)	0.379 (0.343–0.405)	−0.469	0.693
MD (10^−3^ mm^2^/s)	0.768 (0.674–0.932)	0.620 (0.560–0.665)	−3.620	<0.001*	0.636 (0.607–0.672)	0.614 (0.593–0.669)	−1.735	0.083
RD (10^−3^ mm^2^/s)	0.629 (0.549–0.828)	0.499 (0.446–0.567)	−3.490	<0.001*	0.503 (0.469–0.535)	0.489 (0.455–0.547)	−1.063	0.288
AK	0.666 (0.592–0.695)	0.753 (0.712–0.761)	−4.042	<0.001*	0.724 (0.689–0.762)	0.752 (0.732–0.768)	−2.484	0.013*
MK	0.796 (0.678–0.825)	0.915 (0.877–0.954)	−3.945	<0.001*	0.895 (0.856–0.932)	0.924 (0.891–0.956)	−2.475	0.013*
RK	0.933 ± 0.155	1.115 ± 0.139	−5.578	<0.001*	1.114 (1.041–1.178)	1.152 (1.068–1.199)	−2.054	0.040*
MSD (10^−5^ mm^2^/s)	18.634 (17.640–23.019)	16.612 (15.271–18.092)	−2.734	0.006*	16.713 (15.672–17.609)	16.412 (15.715–17.927)	−0.779	0.436
NG	0.230 (0.194–0.253)	0.267 (0.262–0.274)	−3.750	<0.001*	0.269 (0.252–0.279)	0.272 (0.263–0.279)	−1.236	0.271
NGAx	0.197 (0.162–0.219)	0.227 (0.221–0.234)	−3.036	0.002*	0.228 (0.214–0.237)	0.231 (0.222–0.239)	−0.788	0.431
NGRad	0.116 (0.097–0.131)	0.141 (0.135–0.146)	−3.782	<0.001*	0.143 (0.132–0.153)	0.146 (0.139–0.160)	−1.210	0.226
QIV (10^−5^ mm^2^/s)	33.644 (23.000–56.418)	19.050 (15.820–24.928)	−3.328	<0.001*	20.184 (16.800–25.140)	18.851 (16.673–24.493)	−1.244	0.231
RTAP (10^−5^ mm^2^/s)	5.579 ± 1.072	7.729 ± 1.040	−5.169	<0.001*	7.050 (6.488–7.552)	7.233 (6.651–7.851)	−31.649	0.099
RTOP (10^−5^ mm^2^/s)	3.860 (3.218–4.443)	5.285 (4.606–5.745)	−3.620	<0.001*	5.069 ± 1.029	5.219 ± 0.707	−1.379	0.174
RTPP (10^−5^ mm^2^/s)	5.165 ± 0.308	5.510 ± 0.198	−4.453	<0.001*	5.405 ± 0.286	5.500 ± 0.168	−1.674	0.100
ICVF	0.476 (0.397–0.508)	0.582 (0.554–0.602)	−4.107	<0.001*	0.559 (0.534–0.588)	0.584 (0.568–0.609)	−3.018	0.003*
ISOVF	0.253 (0.216–0.328)	0.227 (0.183–0.287)	−1.308	0.168	0.231 (0.208–0.262)	0.234 (0.209–0.265)	−0.392	0.695
ODI	0.322 ± 0.079	0.341 ± 0.065	−0.642	0.528	0.319 ± 0.053	0.334 ± 0.047	−1.652	0.104

MW, motor weakness; NM, normal motor; CST, corticospinal tract. AD, axial diffusivity; FA, fractional anisotropy; MD, mean diffusivity; RD, radial diffusivity. AK, axial kurtosis; MK, mean kurtosis; RK, radial kurtosis. MSD, mean squared displacement; NG, non-Gaussianity; NGAx, NG axial; NGRad, NG radial; QIV, Q-space inverse variance; RTAP, return to the axis probability; RTOP, return to the origin probability; RTPP, return to the plane probability; ICVF, intracellular volume fraction; ISOVF, isotropic volume fraction; ODI, orientation dispersion index. *p*-values were adjusted for multiple comparisons using the Benjamini-Hochberg procedure. *p < 0.05 was considered statistically significant.

### Comparison of relative CST diffusion parameters between MW and NM groups

**Figure 1:**
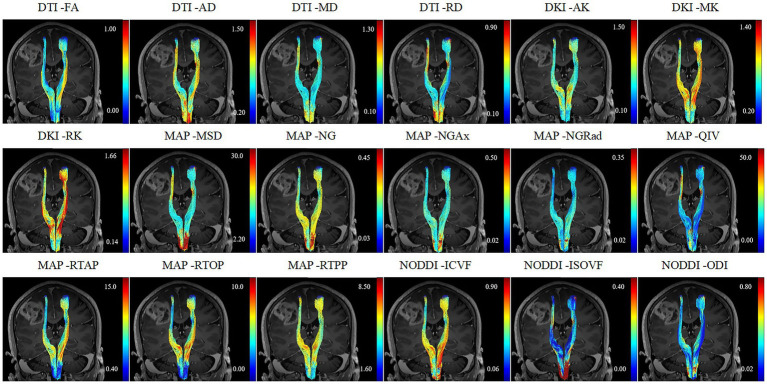
A 52-year-old female patient diagnosed with glioblastoma and presented with motor weakness.

**Figure 2:**
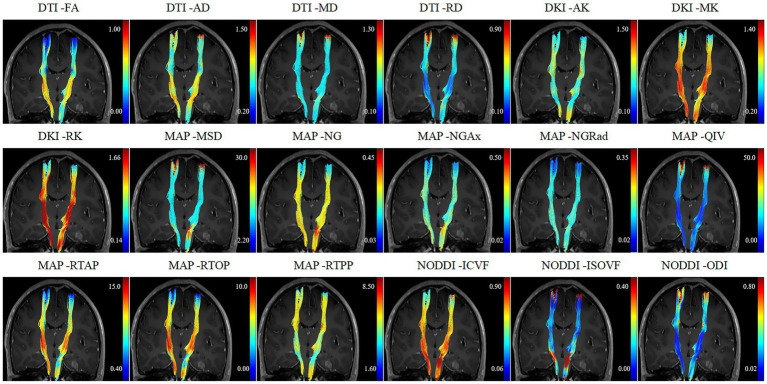
A 57-year-old female patient diagnosed with glioblastoma presented with normal motor.

All relative CST diffusion parameters were significantly different between the MW group and NW group, except for relative AD (*p* = 0.119), relative RTPP (*p* = 0.128), relative ISOVF (*p* = 0.131), and relative ODI (*p* = 0.162). Compared to the NW group, the MW group had significantly higher relative MD, relative RD, relative MSD, relative QIV (all *p* < 0.01), and significantly lower relative FA, relative AK, relative MK, relative RK, relative NG, relative NGAx, relative NGRad, relative RTAP, relative RTOP, relative ICVF (all *p* < 0.01). After the adjustment for multiple comparisons, the relative diffusion parameters remained significantly statistical difference between two groups, except for relative AD, RTPP, ISOVF and ODI. Table 2 presents detailed results. Figures 1, 2 depict data from two representative patients, emphasizing the distinctions in CST characteristics between patients with motor weakness and those with normal motor function.

**Table 2:** Comparison of relative CST diffusion parameters between MW and NM groups.

Relative CST diffusion parameters	MW (*n* = 22)	NM (*n* = 54)	*z*	*p*
AD (10^−3^ mm^2^/s)	1.714 (0.573–2.247)	1.022 (0.990–1.084)	−1.558	0.119
FA	0.849 (0.676–1.005)	1.005 (0.931–1.099)	−2.944	0.003*
MD (10^−3^ mm^2^/s)	1.196 (1.040–1.511)	1.05 (0.970–1.012)	−3.459	<0.001*
RD (10^−3^ mm^2^/s)	1.231 (1.071–1.656)	1.020 (0.946–1.100)	−3.780	<0.001*
AK	0.896 (0.786–0.952)	0.971 (0.920–1.017)	−3.573	<0.001*
MK	0.854 (0.737–0.925)	0.981 (0.929–1.022)	−4.261	<0.001*
RK	0.810 (0.660–0.893)	0.973 (0.920–1.014)	−4.146	<0.001*
MSD (10^−5^ mm^2^/s)	1.121 (1.026–1.356)	1.007 (1.064–1.11)	−3.482	<0.001*
NG	0.854 (0.721–0.935)	0.971 (0.928–1.031)	−3.803	<0.001*
NGAx	0.871 (0.731–0.948)	0.966 (0.930–1.014)	−2.863	0.004 *
NGRad	0.802 (0.749–0.944)	0.957 (0.865–1.048)	−2.199	0.028 *
QIV (10^−5^ mm^2^/s)	1.764 (1.103–3.234)	1.022 (0.870–1.317)	−3.241	0.001*
RTAP (10^−5^ mm^2^/s)	0.794 (0.595–0.929)	0.982 (0.895–1.036)	−3.447	<0.001*
RTOP (10^−5^ mm^2^/s)	0.826 (0.631–0.906)	0.973 (0.892–1.050)	−3.173	0.002 *
RTPP (10^−5^ mm^2^/s)	0.958 (0.897–1.008)	0.989 (0.963–1.010)	−1.523	0.128
ICVF	0.776 (0.651–0.901)	0.963 (0.886–0.999)	−4.261	<0.001*
ISOVF	1.030 (0.911–1.341)	0.978 (0.871–1.097)	−1.512	0.131
ODI	0.873 (0.780–1.040)	0.939 (0.869–1.032)	−1.397	0.162

MW, motor weakness; NM, normal motor; CST, corticospinal tract. AD, axial diffusivity; FA, fractional anisotropy; MD, mean diffusivity; RD, radial diffusivity. AK, axial kurtosis; MK, mean kurtosis; RK, radial kurtosis. MSD, mean squared displacement; NG, non-Gaussianity; NGAx, NG axial; NGRad, NG radial; QIV, Q-space inverse variance; RTOP, return to the origin probability; RTAP, return to the axis probability; RTPP, return to the plane probability; ICVF, intracellular volume fraction; ISOVF, isotropic volume fraction; ODI, orientation dispersion index. *p*-values were adjusted for multiple comparisons using the Benjamini-Hochberg procedure. *p < 0.05 was considered statistically significant.

### Comparing the performance of relative CST diffusion parameters in evaluating the CST injury

**Figure 3:**
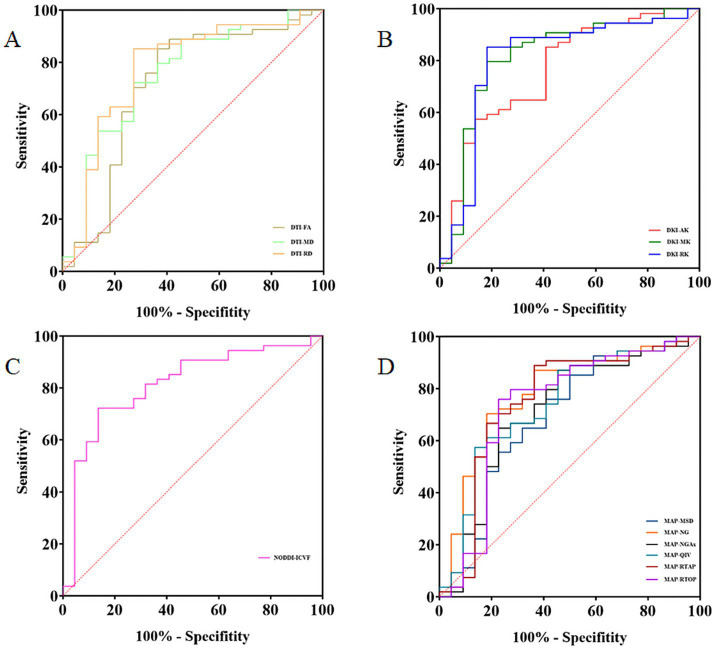
The receiver operating characteristic (ROC) analysis of the relative CST diffusion parameters. ROC curves of the relative CST diffusion parameters based on DTI, DKI, NODDI, and MAP-MRI **(A–D)**.

ROC curve analyses were conducted to assess the performance of the relative CST diffusion parameters in the MW and NM groups. Table 3 and Figure 3 shows the ROC analyses of the significant diffusion parameters. Notably, relative MK and ICVF attained the highest area under the curve (AUC) (AUC = 0.813). Relative MK demonstrated a sensitivity of 81.82% and specificity of 79.63%, while relative ICVF showed a sensitivity of 86.36% and specificity of 72.22%. Relative RK achieved the second-highest AUC (AUC = 0.805), with a sensitivity of 81.82% and specificity of 85.19%. Relative NG ranked third in terms of AUC (AUC = 0.779), with a sensitivity of 81.82% and specificity of 70.37%. Relative RD had the fourth-highest AUC (AUC = 0.778), demonstrating a sensitivity of 72.73% and specificity of 85.19%. Notably, the relative MK parameter exhibited significantly enhanced performance compared to the relative RD (Table 4).

**Table 3:** The performance of relative CST diffusion parameters in evaluating the CST injury.

Relative CST diffusion parameters	AUC (95% CI)	Cut-off value	Sensitivity (%)	Specificity (%)
FA	0.716 (0.601–0.814)	0.488	63.64	85.19
MD (10^−3^ mm^2^/s)	0.754 (0.642–0.846)	0.450	72.73	72.22
RD (10^−3^ mm^2^/s)	0.778 (0.668–0.865)	0.5791	72.73	85.19
AK	0.763 (0.651–0.853)	0.443	59.09	85.19
MK	0.813 (0.707–0.893)	0.615	81.82	79.63
RK	0.805 (0.698–0.887)	0.670	81.82	85.19
MSD (10^−5^ mm^2^/s)	0.756 (0.644–0.847)	0.443	59.09	85.19
NG	0.779 (0.670–0.867)	0.522	81.82	70.37
NGAx	0.710 (0.595–0.809)	0.421	77.27	64.81
QIV (10^−5^ mm^2^/s)	0.738 (0.625–0.832)	0.438	86.36	57.41
RTAP (10^−5^ mm^2^/s)	0.753 (0.641–0.845)	0.525	63.64	88.89
RTOP (10^−5^ mm^2^/s)	0.733 (0.619–0.828)	0.532	77.27	75.93
ICVF	0.813 (0.707–0.893)	0.4545	86.36	72.22

AUC, area under the curve; CST, corticospinal tract. FA, fractional anisotropy; MD, mean diffusivity; RD, radial diffusivity. MK, mean kurtosis; RK, radial kurtosis. MSD, mean squared displacement; NG, non-Gaussianity; NGAx, NG axial; QIV, Q-space inverse variance; RTAP, return to the axis probability; RTOP, return to the origin probability; ICVF, intracellular volume fraction.

**Table 4:** Comparing the performance of relative CST diffusion parameters in evaluating the CST injury.

Relative CST features	DeLong’s test (*p* value)
RD vs. MK	0.031*
RD vs. NG	0.930
RD vs. ICVF	0.237
MK vs. NG	0.059
MK vs. ICVF	1.000
NG vs. ICVF	0.161

CST, corticospinal tract. RD, radial diffusivity. MK, mean kurtosis; RK, radial kurtosis. NG, non-Gaussianity. ICVF, intracellular volume fraction. *p < 0.05.

### Subgroup analysis

There was no significant difference in CST displacement between the MW (*n* = 22) and NM (*n* = 37) groups in the subgroup analysis. The results of subgroup analyses were similar to those of the overall analysis. The performance of relative CST diffusion parameters in evaluating CST injury was as follows: relative MK (AUC = 0.800, sensitivity of 81.82%, specificity of 75.68%), relative RK (AUC = 0.795, sensitivity of 81.82%, specificity of 81.08%), relative ICVF (AUC = 0.785, sensitivity of 86.36%, specificity of 64.86%), relative RD (AUC = 0.764, sensitivity of 72.73%, specificity of 81.08%), relative NG (AUC = 0.756, sensitivity of 63.64%, specificity of 83.78%), in descending order. Furthermore, the efficiency of the relative MK was better than that of the relative RD. Detailed results of the subgroup analyses are presented in Supplementary Tables S2–S4.

### Correlation analysis between the relative MK parameter and muscle strength

**Figure 4:**
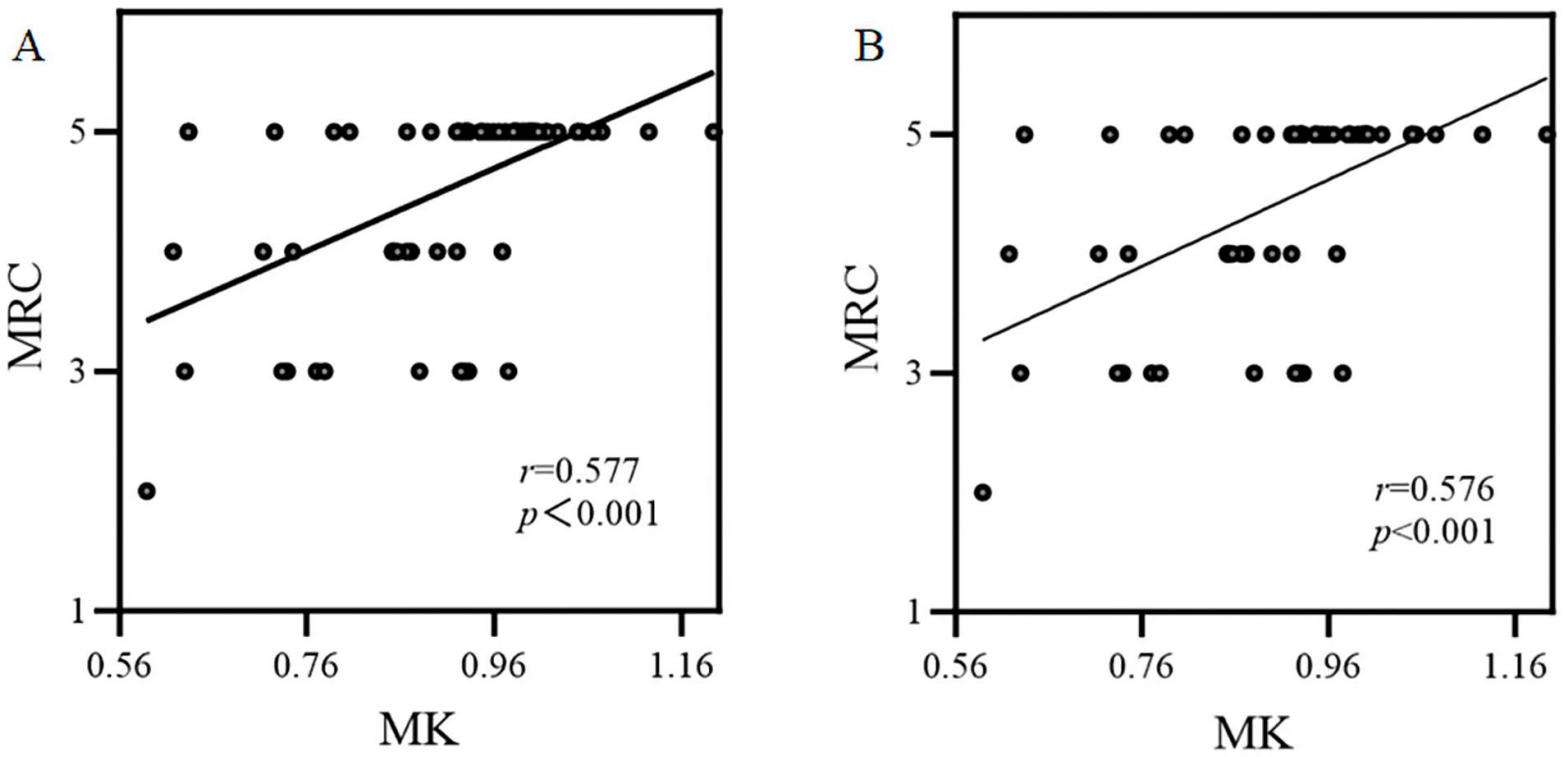
The correlation between the relative MK parameter and muscle strength assessed by MRC. **(A)** Overall analysis; **(B)** Subgroup analysis.

As Figure 4 shows, in 76 patients with GBM, the relative MK value in CST was significantly positively correlated with the MRC (*r* = 0.577, *p* < 0.001). The results of subgroup analyses were similar to those of the overall analysis (*r* = 0.576, *p* < 0.001).

## Discussion

GBM is the most common primary malignant tumor in adults, characterized by diffuse infiltration of surrounding white matter tracts (Lapointe et al., 2018). This study aimed to explore the application of multiple diffusion models––DTI, DKI, NODDI, and MAP in assessing CST injury induced by GBM while also comparing their performance. Our findings suggest that diffusion parameters obtained from DTI, DKI, NODDI, and MAP can effectively evaluate corticospinal tract injury. Among these models, DKI-based relatives MK demonstrated superior performance, exhibiting heightened sensitivity and specificity, and which significantly positively correlated with muscle strength.

Compared to the healthy CST, the diffusion parameters of the affected CST were altered, regardless of whether they were in the MW or NM group. These changes can be attributed to the invasive nature of GBM, which tends to invade existing extracellular structures. Previous studies have indicated that GBM typically utilizes nerve fibers as a guide for infiltration, leading to abnormalities in the microstructure of these fibers (El Ouadih et al., 2022; Li et al., 2023), including those within the CST. Specifically, in the MW group, all CST diffusion parameters (except for NODDI-based ISOVF and ODI) showed significant differences between the affected and healthy sides. In the NM group, DKI-based AK, MK, and RK exhibited significant differences between the affected and healthy sides, while only AD based on DTI and ICVF based on NODDI demonstrated changes. This suggests that the relevant DKI-derived parameters are more sensitive in detecting microstructural alterations compared to other diffusion parameters such as FA, MD, and so on.

Quantitative assessment of white matter tracts remains a significant challenge. Previous DTI studies on aging have reported an increase in MD and a decrease in FA with age, alongside significant individual variability (Bennett et al., 2010; Molloy et al., 2021). Therefore, we employed relative CST diffusion parameters in this study to mitigate the effects of age and inter-individual differences (Min et al., 2017). The results revealed that the MW group exhibited significantly heightened relative MD, RD, MSD, and QIV, along with diminished relative FA, AK, MK, RK, NG, NGAx, NGRad, RTAP, RTOP, ICVF compared to the NW group. It is worth noting that there was no statistical difference in terms of relative ISOVF and ODI between the MW group and the NW group. ISOVF – a metric derived from an isotropic diffusion signal from the cerebrospinal fluid (CSF) compartment, reflecting CSF-like free water diffusion. The absence of significant intergroup differences in ISOVF may suggest that GBM-induced CST damage primarily alters axonal integrity rather than expanding extracellular free water compartments (Nemanich et al., 2019). ODI reflects the spread of neurite orientation, which effectively captures neurite dispersion in crossing or dispersed fibers. However, its utility in assessing highly aligned white matter such as the CST may be constrained, where inherently low orientation dispersion limits the discriminatory power (Zhang et al., 2012). GBM may only cause minor changes in dispersion of CST. Critically, the displacement of the CST constituted a significant confounding factor. Patients with motor weakness exhibited substantially greater displacement distances than those with normal motor function, suggesting that the greater the displacement distance, the higher probability of CST damage. To eliminate the influence of this confounding factor, we further selected cases of glioblastoma with significant displacement of the corticospinal tract for subgroup analysis, in order to balance the two groups. The subgroup analysis results aligned with the overall study, showing that relative MK and RK had higher AUCs, demonstrating higher sensitivity and specificity. Furthermore, the performance of the relative MK was superior to that of the relative RD. These findings suggest that DKI offers greater promise in assessing microstructural alterations in CST injury than traditional DTI metrics.

The performance of the advanced diffusion model, DKI, in evaluating CST damage was better than that of DTI, potentially attributed to the inherent limitations of DTI. The actual brain microstructure is complex and does not follow a Gaussian distribution. DKI specifically takes into account the complexity of brain microstructure by assessing kurtosis (Wu and Cheung, 2010). MK, which represents the average kurtosis across all diffusion directions, reflects microstructural complexity, with reduced values indicating compromised cellular integrity and enhanced membrane permeability (Steven et al., 2014; Lee et al., 2013). RK, measuring the kurtosis along the radial direction and higher in white matter, is sensitive to the integrity of myelin and cellular membranes (Raab et al., 2010). A decline in RK indicates impaired myelin and cell membrane integrity (Oliviero and Del Gratta, 2021; Rosenkrantz et al., 2015). In cases where GBM invades the CST pathway, impairing diffusion barriers and decreasing microstructural complexity, there are marked decreases in MK and RK. Additionally, in recent studies, NODDI and MAP have been used to assess CST injury in glioma, achieving performance comparable to that of DTI (Jiang et al., 2021; Jiang et al., 2021), which is consistent with our results. Although based on technical principles, NODDI and MAP may be able to provide more abundant information on the CST than DTI and DKI, these advantages are on the basis of indirect results lacking further histological validation (Gao et al., 2022). Moreover, while DKI-based MK and NODDI-based ICVF obtained identical AUC value in our results, the Youden index of MK is higher than that of ICVF (0.614 vs. 0.585), suggesting its more balanced sensitivity-specificity profile may better meet clinical requirements. Our results did not demonstrate the superiority of MAP over DTI; however, RTAP based on MAP demonstrated the highest specificity. RTAP is a parallel scalar along the white matter tract, which reflects the restrictive barriers in the radial orientation (Ozarslan et al., 2013; Fick et al., 2016). As mentioned above, CST injury may lead to the destruction of diffusion barriers, and our findings revealed that RTAP, as the radial component of MAP, may have a high capability to detect white matter tract damage, providing additional insights into understanding CST injury. Given the more balanced performance of DKI, DKI-derived metrics enable precise preoperative mapping of CST integrity loss near tumors. By integrating these quantitative parameters into intraoperative navigation systems surgeons can dynamically adjust resection margins to spare functionally critical fibers, reducing iatrogenic motor deficits.

This study has some limitations. First, a recognized shortcoming is the absence of a uniform protocol for tracking white matter tracts, and the impact of mass effects including white matter displacement, edema-induced diffusion alterations, and tumor infiltration, which may prematurely terminate streamlines or distort local orientation estimates. We chose the advanced tractography with GQI algorithm to map white matter tracts more accurately, complete CST visualization remains challenging in severe mass effect scenarios. Second, our study lacks a longitudinal analysis of the surgical impact on motor function in patients with GBM, a critical aspect that should be addressed in future studies. Third, our sample size was somewhat limited; however, it is important to note that we exclusively selected patients with GBM, in contrast to previous studies encompassing various types of brain tumors. Future studies should aim to recruit a larger population for a more comprehensive investigation. Finally, acquiring advanced model parameters requires specialized post-processing software, which may be inconvenient for clinical applications; however, future developments in the production and automation of post-processing technology are anticipated to solve this limitation.

## Conclusion

In conclusion, diffusion parameters derived from DTI, DKI, NODDI, and MAP prove effective in assessing CST injury. While DKI-derived MK and NODDI-derived ICVF achieved identical high AUC values, MK exhibited a more balanced sensitivity-specificity profile for assessing microstructural alterations in CST injury, this advantage of DKI may better address clinical demands, potentially aiding in surgical planning and preserving motor function in patients with GBM.

## Data Availability

The original contributions presented in the study are included in the article/Supplementary material, further inquiries can be directed to the corresponding authors.

## Footnotes

^1^ https://diffusionkit.readthedocs.io/

^2^ https://www.dipy.org

^3^ http://dsi-studio.labsolver.org
