# Supplementary material for: Utilizing multiple diffusion metrics in evaluation of corticospinal tract injury in patients with glioblastoma
Source: Front Neurosci. 2025 Jul 28;19:1605786. doi: 10.3389/fnins.2025.1605786 (PMC12336199; doi:10.3389/fnins.2025.1605786)
Supplement: Supplementary file 1 [file Data_Sheet_1.docx]

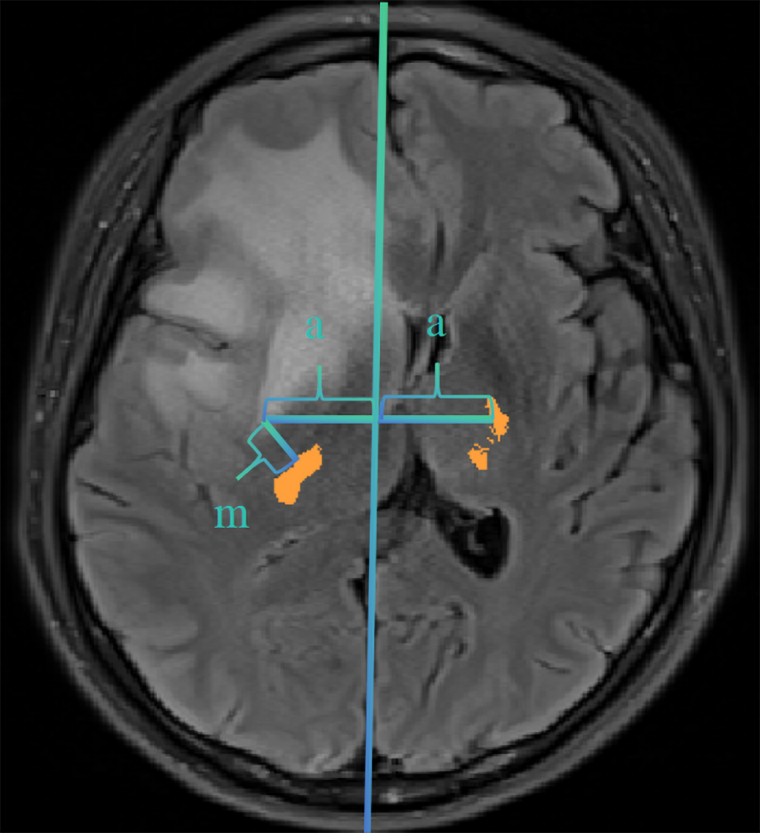


Fig. S1 Measurement displacement of the affected CST. The distance between the symmetrical position of the healthy and actual position was used to quantify the displacement of affected CST (m). The healthy CST and its symmetrical position have the same vertical distance to the midline (a).
