# Supplementary material for: Utilizing multiple diffusion metrics in evaluation of corticospinal tract injury in patients with glioblastoma
Source: Front Neurosci. 2025 Jul 28;19:1605786. doi: 10.3389/fnins.2025.1605786 (PMC12336199; doi:10.3389/fnins.2025.1605786)
Supplement: Supplementary file 2 [file Table_1.docx]

**TABLE S1.** **Clinical characteristics of participants**

| Variables | MW(n=22) | NM (n=54) | *P* |
| --- | --- | --- | --- |
| Age (years) | 54.50(52.00-65.25) | 55.00(48.75-63.25) | 0.477 |
| Sex |  |  | 0.566 |
| Male | 13(59.10%) | 28(51.90%) |  |
| Female | 9(40.90%) | 26(48.10%) |  |
| KPS | 70.00(50.00-80.00) | 90.00(80.00-90.00) | <0.001﹡ |
| Tumor location |  |  | 0.051 |
| Frontal&Parietal lobe | 16(72.70%) | 26(51.90%) |  |
| Other location | 6(27.70%) | 28(48.10%) |  |
| Tumor size (cm^3^ ) | 56.65(26.22-98.77) | 36.85(18.43-83.92) | 0.196 |
| Positional relationship |  |  | 0.116 |
| In the CST pathway | 21(95.50%) | 44(81.50%) |  |
| Near the CST pathway | 1(4.50%) | 10(18.50%) |  |
| CST displacement (mm) | 8.68(10.36-13.28) | 6.50(4.87-10.00) | <0.001﹡ |

MW=motor weakness; NM=normal motor; KPS=Karnofsky performance status; CST=corticospinal tract

**TABLE S2. Comparison of** **relative CST** **diffusion parameters between MW and NM groups**

| Relative CST features | MW (n=22) | NM (n=37) | z | *P* |
| --- | --- | --- | --- | --- |
| AD(10^-3^mm^2^/s) | 1.714(0.573-2.247) | 1.059(0.988-1.010) | -1.395 | 0.163 |
| FA | 0.849(0.676-1.005) | 1.025(0.927-1.104） | -2.759 | 0.006﹡ |
| MD(10^-3^mm^2^/s) | 1.196(1.040-1.511) | 1.043(0.971-1.105) | -2.978 | 0.003﹡ |
| RD(10^-3^mm^2^/s) | 1.231(1.071-1.656) | 1.023(0.945-1.023) | -3.370 | <.001﹡ |
| AK | 0.874±0.107 | 0.953 ± 0.762 |  | .005﹡ |
| MK | 0.854(0.737-0.925) | 0.950(0.926-1.001) | -3.825 | <0.001﹡ |
| RK | 0.810(0.660-0.893) | 0.971(0.910-1.016) | -3.762 | <0.001﹡ |
| MSD | 1.121(1.026-1.356) | 1.010(0.963-1.062) | -3.213 | 0.001﹡ |
| NG | 0.854(0.721-0.935) | 0.953(0.914-1.032) | -3.260 | 0.001﹡ |
| NGAx | 0.871(0.731-0.948) | 0.965(0.918-1.029） | -2.524 | 0.012﹡ |
| NGRad | 0.802(0.749-0.944) | 0.921(0.854-1.069) | -2.132 | 0.033﹡ |
| QIV(10^-5^mm^2^/s) | 1.764(1.103-3.234) | 1.055(0.866-1.420) | -2.916 | 0.004﹡ |
| RTAP(10^-5^mm^2^/s) | 0.794(0.595-0.929) | 0.961(0.890-1.040) | -3.010 | 0.003﹡ |
| RTOP(10^-5^mm^2^/s) | 0.826(0.631-0.906) | 0.945(0.853-1.06) | -2.806 | 0.005﹡ |
| RTPP(10^-5^mm^2^/s) | 0.958(0.897-1.008) | 0.978(0.958-1.006) | -1.285 | 0.199 |
| ICVF | 0.776(0.651-0.901) | 0.963(0.886-0.999) | -3.637 | <.001﹡ |
| ISOVF | 1.030(0.911-1.341) | 0.978(0.871-1.097) | -1.536 | 0.125 |
| ODI | 0.873(0.780-1.040) | 0.939(0.869-1.032) | -0.799 | 0.424 |

MW=motor weakness; NM=normal motor; CST=corticospinal tract. AD=axial diffusivity; FA=fractional anisotropy; MD=mean diffusivity; RD=radial diffusivity. AK=axial kurtosis; MK=mean kurtosis; RK=radial kurtosis. MSD=mean squared displacement; NG=non-Gaussianity; NGAx=NG axial; NGRad= NG radial; QIV=Q-space inverse variance; RTOP=return to the origin probability; RTAP=return to the axis probability; RTPP=return to the plane probability; ICVF=intracellular volume fraction; ISOVF=isotropic or free water volume fraction; ODI=orientation dispersion index. P-values were adjusted for multiple comparisons using the Benjamini-Hochberg procedure,﹡P < 0.05.

**TABLE S3.** **The performance of relative CST diffusion parameters in evaluating the CST injury**

| Relative CST features | AUC (95% CI) | Cut-off value | Sensitivity (%) | Specificity (%) |
| --- | --- | --- | --- | --- |
| FA | 0.716(0.584-0.826) | 0.488 | 63.64 | 83.78 |
| MD(10^-3^mm^2^/s) | 0.733(0.602- 0.840) | 0.410 | 54.55 | 86.49 |
| RD(10^-3^mm^2^/s) | 0.764(0.636 - 0.865) | 0.538 | 72.73 | 81.08 |
| AK | 0.719(0.586 -0.828) | 0.402 | 59.09 | 81.08 |
| MK | 0.800(0.675- 0.893) | 0.575 | 81.82 | 75.68 |
| RK | 0.795(0.670-0.899) | 0.670 | 81.82 | 81.08 |
| MSD(10^-5^mm^2^/s) | 0.752(0.626-0.858) | 0.439 | 68.18 | 75.68 |
| NG | 0.756(0.670- 0.867) | 0.474 | 63.64 | 83.78 |
| NGAx | 0.698(0.564 -0.811) | 0.383 | 54.55 | 83.78 |
| NGRad | 0.667(0.532 -0.784) | 0.356 | 55.55 | 81.08 |
| QIV(10^-5^mm^2^/s) | 0.729(0.597 - 0.836) | 0.413 | 81.82 | 59.46 |
| RTAP(10^-5^mm^2^/s) | 0.736(0.605-0.842) | 0.528 | 63.64 | 89.19 |
| RTOP(10^-5^mm^2^/s) | 0.720(0.588- 0.829) | 0.484 | 72.73 | 75.68 |
| ICVF | 0.785(0.659- 0.881) | 0.512 | 86.36 | 64.86 |

AUC = area under the curve; CST=corticospinal tract. FA=fractional anisotropy; MD=mean diffusivity; RD=radial diffusivity. AK=axial kurtosis; MK=mean kurtosis; RK=radial kurtosis. MSD=mean squared displacement; NG=non-Gaussianity; NGAx=NG axial; NGRad=NG radial; QIV=Q-space inverse variance; RTAP=return to the axis probability; RTOP=return to the origin probability. ICVF=intracellular volume fraction.

**TABLE S4. Comparing the performance of relative CST diffusion parameters in evaluating the CST injury**

| Relative CST features | DeLong’s test ^a^  (P value) |
| --- | --- |
| RD vs. MK | 0.040﹡ |
| RD vs. NG | 0.692 |
| RD vs. ICVF | 0.497 |
| MK vs. NG | 0.036﹡ |
| MK vs. ICVF | 0.548 |
| NG vs. ICVF | 0.260 |

CST=corticospinal tract. RD=radial diffusivity. MK=mean kurtosis; RK=radial kurtosis. NG=non-Gaussianity; ICVF=intracellular volume fraction.﹡P < 0.05.
